# Assessing the impact of medical studies on students’ motivation, satisfaction, stress and values in Poland: a cross-sectional study

**DOI:** 10.1186/s12909-025-07287-4

**Published:** 2025-05-16

**Authors:** Pola Sarnowska, Julia Terech, Klaudia Bikowska, Mateusz Guziak, Maciej Walkiewicz

**Affiliations:** 1 Faculty of Medicine, Student Scientific Circle of Psychology, Medical University of Gdańsk, Gdańsk, Poland; 2 Department of Psychology, Faculty of Health Sciences with the Institute of Maritime and Tropical Medicine, Medical University of Gdańsk, Gdańsk, Poland

**Keywords:** Students, medical, Education, medical, Motivation, Personal satisfaction, Stress, psychological, Resilience, psychological, Values, Poland

## Abstract

**Background:**

The demanding nature of the medical career path leads to reflection on the motivations, values and expectations of medical students towards their course, their satisfaction with its components and the stress they experience. Research suggests that these parameters may change in the course of the studies, which may be linked to varying forms of the training and students’ personal circumstances. The following study aimed to analyse differences in these areas across various stages of medical education.

**Methods:**

A total of 334 Polish medical students in 1st, 4th and 6th year were surveyed. The study included questionnaires to assess motivations for choosing medical studies, satisfaction with them, students’ values, competencies developed during the studies and the Brief Resilience Scale. Additional questions addressed students’ life situation and stress levels, preferred medical specialties, and alternative career paths. The Mann-Whitney U test with Benjamini-Hochberg p-value correction was applied to analyse motivation, health and stress levels, and satisfaction. Fisher’s exact test with Benjamini-Hochberg p-value correction was applied to assess life values, competencies, medical specialty preferences and alternative paths.

**Results:**

Significant differences were found in motivations to study such as interests, high income and social prestige, as well as in health assessment and stress levels, satisfaction with the university, relationships with peers and teachers, various classes, practical skills, workload and time spent on studies. Life values showed shifts in the importance of peace and quiet, education, achievement and fame. Competencies gained and expected to develop differed by the year of study. Resilience levels showed no significant changes across the groups. Few notable results were found regarding the changes in specialty preferences or consideration of alternative career paths.

**Conclusions:**

As students advance in their medical education, extrinsic motivations such as financial gain and prestige become less prominent, but intrinsic motivations like interest in the subject also decrease. Satisfaction with medical education diminishes over time, particularly in areas related to the university, relationships, or skills. Values such as achievement, fame and education gradually become less important. Interpersonal and analytical skills appear to develop more prominently in the later stages of training. Stress levels typically peak around the fourth year of study.

**Supplementary Information:**

The online version contains supplementary material available at 10.1186/s12909-025-07287-4.

## Introduction

The medical profession belongs to the most demanding ones. According to law, it is included in the professions of public trust [1], which is inseparably linked to high social requirements towards doctors, especially in the area of doctor-patient communication. In their cross-sectional study of the changes of values among students of medical schools, Gordon and Mensh state that there is a downward trend in benevolence and conformity between the first and the fourth year of medical education, which they speculate may be linked to an increase in problem orientation and a decrease in social orientation [2]. The data indicate that the values of medical trainees may not only be far from social expectations, but they also shift and change in the course of medical education [3], which may be due to the shift in the form of the training (from theoretical to practical, from preclinical to clinical) over the years.

The topic of motivation of medical students is explored through numerous research. In this article, motivation will be defined as ‘the process whereby goal-directed activities are initiated and sustained’ [4]. Therefore, it enables meeting both one’s own and imposed requirements. A well-established one is the basis for engagement in the activity being performed [5]. According to research conducted at Polish universities, the predominant motives for taking up medical studies are the desire to help, a chance to find employment after graduation, the high prestige of the profession, and the fulfilment of aspirations [1, 5, 6]. Interestingly enough, motivation evolves over the course of medical education due to various factors such as academic pressure, exposure to clinical environments, and personal life changes. Del-Ben et al. [7] present a significant decline in intrinsic motivation during the first year of medical studies. Likewise, research by Kusurkar et al. highlights that intrinsic motivation tends to decline over time while extrinsic motivations, such as financial rewards or societal status, may increase [8]. The shift in motivation during medical education can influence long-term career satisfaction and suggests that satisfaction and a sense of fulfilment may be primary drivers for pursuing medical studies. The beginning of a clinical course can reignite students’ passion, but it also introduces significant emotional and physical demands.

The nature of the medical field leads to reflection not only on the motivation but also on students’ satisfaction with this particular career path. Ziaee et al. in their study evaluating students’ satisfaction with clinical education during internship show that overall satisfaction was on the level of mere 38.8%, with 52% of the 250 respondents being satisfied with bedside teaching and 70.8% - with theoretical education [9]. These findings indicate that there may be a difference between satisfaction with theoretical and practical course aspects depending on various circumstances on an organisational or personal level. Eyck et al. emphasise the importance of simulation-based learning in their research on students’ satisfaction and test performance when using a simulation-based emergency medicine curriculum as opposed to group discussion [10]. This poses the question of whether course satisfaction increases with increasing exposure of the student to real-life-like problem-solving. In addition to that, students’ satisfaction with their professional accomplishments tends to increase in the course of their education, even though their tendency to experience burnout syndrome, which interferes with job performance, also increases [11]. The topic of satisfaction needs to be studied further in terms of factors affecting it on different levels and in different forms of medical education, as well as the ones resulting from the personal circumstances of the students involved.

The work of a medical professional is mentally burdensome due to constant stress, daily contact with suffering patients, or constant exposure to the risk of losing health or life [12]. Medical students list stressors such as concern about the future, defective skills, time constraints and fear of doing harm to patients [13]. An individual’s ability to cope with these threats is a matter of resilience. In psychology, resilience is defined as the ability to adapt to unfavourable circumstances and to recover from experienced stress [14]. It can play an important role in preventing burnout and mitigating the consequences of stress, which is a part of the everyday life of medical students. As research provides us with evidence that stress levels fluctuate during the medical course, it can be hypothesised that resilience may fluctuate, too. However, findings are not consistent when determining the direction of stress levels’ changes. While some researchers claim that first-year students experience more stress [15], others prove that students starting clinical classes are the ones more stressed than their younger colleagues [16]. Findings on medical students’ health appear to be similar - there is evidence that health problems are more pronounced in younger students [17], but at the same time there are implications that health assessment is lower in older students [18]. In addition to that, medical students are prone to experiencing imposter feelings [19], which seem to peak around the 3rd year of medical education [20] and are associated with burnout indices [21]. However, Wolf and Kissling claim that despite the increasing stress level during the first year of studies, students tend to improve their coping strategies [17]. The latter may suggest that there is an increase in resilience as well, yet these findings still need to be supported by further research, including studies conducted in Polish conditions.

Medical studies in Poland takes six years, with the initial three dedicated primarily to theoretical learning, followed by three clinical years that emphasise hands-on experience with patients, allowing students to develop essential practical skills. Recently, the rising demand for medical professionals has led to the creation of numerous private institutions offering medical education. As a result, admission criteria have become somewhat less stringent, making it easier for prospective students to enter medical programs compared to previous years. This shift reflects a response to the healthcare system’s need for more practitioners, though it also raises concerns about maintaining high educational standards. There is also a notable absence of mechanisms to revalidate the medical curriculum, as students’ feedback on their satisfaction with their studies is rarely solicited. Consequently, students may feel uncertain about their career path because they receive little guidance on post-graduation steps, such as internships and specialties. Furthermore, the curriculum tends to overload students with extensive content, including highly specialised information that is only applicable to certain fields, while neglecting essential core competencies that every medical professional should master. This creates a gap in practical knowledge and leads to inefficiencies in preparing students for the realities of their medical careers [22].

The need to investigate this subject is clear, particularly in light of the evident research gap within the Polish context. While international studies offer valuable insights into medical students’ motivation, satisfaction, stress, and resilience, there is a lack of comprehensive research that addresses these issues specifically in Poland. Conducting new research in this country would provide evidence-based recommendations with direct practical implications for enhancing the quality of medical education and improving the support systems for students.

### Purpose of the study

**Fig. 1:**
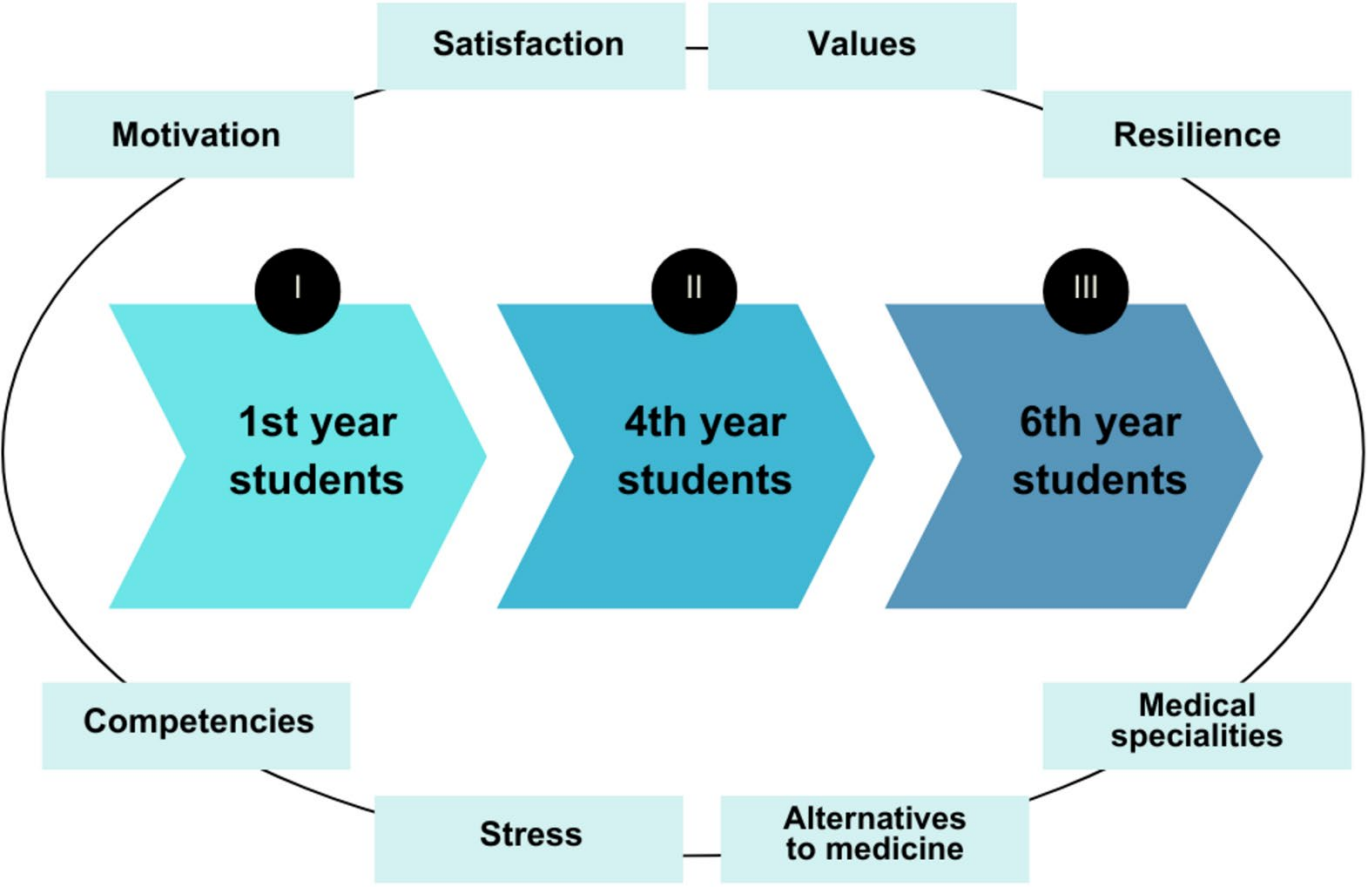
Model of the research

The aim of the study was to analyse psychological and motivational differences among medical students at different stages of their education (Fig. 1). The study addresses the following research questions:

1. Do motivation, satisfaction, value system, resilience, developed and expected competencies differ among medical students at different stages of their education? If yes, in what ways?
2. Do students at different levels of education differ in the stress they experience? If yes, in what ways?
3. How do medical students at different stages of education perceive their future career paths, including their choice of medical specialties and consideration of alternative professional directions?

## Methods

### Study measures

The survey included six questionnaires. Items addressing sociodemographic variables and selected aspects of medical studies (such as year of study or the participant’s university) were followed by 4 items dedicated to self-reported assessment of personal circumstances, which included an evaluation of the participant’s financial situation, state of health, general life satisfaction and current stress levels associated with medical studies (all rated on a 7-Likert scale).

Motivation for medical studies was measured by a questionnaire (10 items in total, 9 of which required a response on a 7-Likert scale and 1 was an open-ended question) prepared by the research team based on a Polish literature review [1, 5, 6, 23, 24]. In the post-factum validation of motivation questionnaire authors obtained the Cronbach-alpha coefficient α = 0.54 and the McDonald’s-omega reliability coefficient ω = 0.13. Further analysis indicated that internal consistency could be improved– after removing two items authors obtained the Cronbach-alpha coefficient α = 0.62 and the McDonald’s-omega reliability coefficient ω = 0.6. Full details are presented in Supplementary Material 2.

A similar method was used to create a questionnaire of satisfaction with medical studies (19 items, responses on a 7-Likert scale) - authors constructed the questions based on previous research [25, 26]. In the post-factum validation of satisfaction questionnaire authors obtained the Cronbach-alpha coefficient α = 0.90 and the McDonald’s-omega reliability coefficient ω = 0.92. Full details are presented in Supplementary Material 3.

In order to describe the participants’ value systems, the authors used a list of 16 values created by the Public Opinion Research Centre (CBOS) and published in 2020 [27]. Students were allowed to choose up to 3 items that corresponded to the most important values in their lives.

Brief Resilience Scale [14] was used to describe participants’ mental resilience. BRS includes 6 items, each one requiring a response on a 5-Likert scale. This standardised questionnaire is based on the concept of individual resilience formulated by Smith et al. [14], according to which individual resilience is the ability to bounce back from a stressful situation. In the current study authors used a validated Polish version of BRS [28].

Competencies developed by students during their medical education were measured by a list of 8 competence categories (participants could choose up to 3). These items were selected based on the definitions of The Great Eight Competencies [29].

At the end of the survey, students were asked about their preferred medical career paths (medical specialties) and what alternatives to studying medicine they would see themselves in. Items addressing assessment of personal circumstances, motivation for medical studies and satisfaction with medical studies are presented in Supplementary Materials 1–3. Supplementary Materials 2, 3 include post-factum validation of motivation and satisfaction questionnaires.

### Data collection

The study was conducted online via Google Forms. The respondents were recruited by social media platforms and medical universities’ internal e-mail boxes. Fluent knowledge of the Polish language and being a student of the 1st, 4th or 6th year of medical studies were the inclusion criteria. The study excluded students participating in English-language programs (such as English Division or Erasmus exchange students), individuals enrolled in non-medical degree programs, medical students outside the designated years of study, and those without reliable internet access.

### Participants

A total of 334 Polish medical students aged 18 to 33 (*M* = 22.26, *SD* = 2.63) were surveyed, 21% (*n* = 71) of them were male and 79% (*n* = 263) were female. Most of the participants were from cities up to 100 000 inhabitants (33%, *n* = 109) When the study was performed, there were approximately 36 000 medical students in Poland [30]. The gender characteristics of the Polish medical students population differed when compared to the studied group, as in the former female students made up 64,4% and male students - 35,6% [30].

Students of the 1st year made up 36% (*n* = 119) of the studied group, 35% (*n* = 118) were 4th-year students and 29% (*n* = 97) − 6th-year students. Most of them declared being Medical University of Gdansk (35%, *n* = 117) and Poznan University of Medical Sciences (26%, *n* = 87) students. Full demographic characteristics of participants are presented in Supplementary Material 4.

### Statistical analysis

Descriptive statistics were used to describe the participants’ sociodemographic characteristics. The mean score and standard deviation of participants’ age and number of their friends were calculated. Other sociodemographic characteristics were expressed in percentage.

Whenever multiple comparisons occurred, Benjamini-Hochberg p-value correction was applied. *P* < 0.05 was considered statistically significant.

## Results

### Motivation to study medicine

A Mann-Whitney U test was performed to evaluate whether motivation to study medicine differed by the year of study (Table 1). Due to multiple comparisons, Benjamini-Hochberg p-value correction was applied.

**Table 1:** Differences in motivation to study between 1st, 4th and 6th -year students

Motivation	Median (IQR)			*U*	*p*
	1st year (*n* = 119)	4th year (*n* = 118)	6th year (*n* = 97)		
My interests	7 (6–7)		6 (5–7)	6942	0.019*
High income in the future	6 (5–7)	5 (5–6)		8396	0.022*
Social prestige	5 (3–6)	5 (3–6)		8268	0.050*

* *p* < 0.05, ** *p* < 0.01, *** *p* ≤ 0.001, IQR - interquartile range

It was found that 1st -year students rated their interests as a motivation for studying medicine significantly higher than 6th -year students, *U* = 6942, *p* = 0.019. Furthermore, when compared to 4th -year students, 1st-year students were significantly more often motivated by high income in the future, *U* = 8396, *p* = 0.022, and also by social prestige of the medical profession, *U* = 8268, *p* = 0.05.

The results indicated that there were no significant differences between the studied groups in rating a clear and secure future, knowledge useful in private life, the fact that their family members worked in health care, a desire to feel needed by others, their conscious decision and a decision made due to others’ expectations as their motivation to study medicine.

### Satisfaction with studying medicine

A Mann-Whitney U test was performed to evaluate whether satisfaction with studying medicine differed by the year of study (Table 2). Due to multiple comparisons, Benjamini-Hochberg p-value correction was applied.

**Table 2:** Differences in satisfaction with studying between 1st, 4th and 6th -year students

Satisfaction component	Median (IQR)			*U*	*p*
	1st year (*n* = 119)	4th year (*n* = 118)	6th year (*n* = 97)		
Overall satisfaction with medical studies	6 (5-6.5)	5 (3–6)		8894.5	0.000***
	6 (5-6.5)		5 (2–6)	8668	0.000***
		5 (3–6)	5 (2–6)	7233.5	0.001***
The mere fact of being admitted to medical studies	7 (6–7)	6.5 (6–7)		8415.5	0.008**
Studying at current university	6 (6–7)	6 (5–7)		8713	0.001***
	6 (6–7)		5 (3–6)	8505	0.000***
		6 (5–7)	5 (3–6)	7199	0.001***
Relationships with other medical students	6 (4–6)		5 (3–6)	6905	0.035*
Relationships with lecturers	5 (4–6)		5 (4–6)	6987	0.009**
		5 (5–6)	5 (4–6)	7149	0.004**
Theoretical classes	5 (4–6)	5 (3–5)		8852.5	0.001***
	5 (4–6)		4 (2–5)	8002.5	0.000***
Practical classes without patients	6 (5–6)	5 (3–6)		9531.5	0.000***
	6 (5–6)		4 (2–5)	8789.5	0.000***
		5 (3–6)	4 (2–5)	7040.5	0.003**
Practical classes with patients		5 (4–6)	5 (3–6)	7124.5	0.002**
Student internships		5 (4–6)	5 (3–6)	6810	0.015*
Practical skills		3 (2–5)	3 (1–5)	6832	0.013*
Amount of learning material	3 (2–5)	4 (3–5)		5687.5	0.031*
		4 (3–5)	3 (2–5)	6679	0.048*
Time spent on studies	3 (2–5)	5 (3–5)		5447	0.004**
		5 (3–5)	3 (2–5)	7048.5	0.004**

* *p* < 0.05, ** *p* < 0.01, *** *p* ≤ 0.001, IQR - interquartile range

The results indicated significant differences in overall satisfaction with medical studies. First-year students were more satisfied than both 4th -year students, *U* = 8894.5, *p* < 0.001, and 6th -year students, *U* = 8668, *p* < 0.001. The last group occurred to be the least satisfied with medical studies (when compared to 4th -year students, *U* = 7233.5, *p =* 0.001). Similarly, 1st -year students were significantly more content with their current university than both 4th -year students, *U* = 8713, *p =* 0.001, and 6th -year students, *U* = 8505, *p* < 0.001. The last group again presented the lowest satisfaction (when compared to 4th -year students, *U* = 7199, *p =* 0.001). What’s more, 1st -year students were significantly more satisfied with the mere fact of being admitted to medical studies when compared to 4th -year students, *U* = 8415.5, *p* = 0.008.

In terms of relationships as a satisfaction component, it was found that 1st -year students were significantly more content with their relationships with other medical students than the 6th -year group, *U* = 6905, *p =* 0.035. What’s more, the results indicated that they were more satisfied with their relationships with lecturers compared to the 6th year, *U* = 6987, *p =* 0.009. Similarly, the last group presented lower satisfaction in this area when compared to 4th -year students, *U* = 7149, *p =* 0.004.

The results showed several differences in satisfaction with classes conducted within the medical studies. The first-year group was significantly more content with theoretical classes than both 4th -year students, *U* = 8852.5, *p =* 0.001, and 6th -year students, *U* = 8002.5, *p* < 0.001; as well as with practical classes without patients (comparing both to 4th year, *U* = 9531.5, *p <* 0.001, and 6th year, *U* = 8789.5, *p* < 0.001). Furthermore, when compared to the 6th -year group, 4th -year students presented a significantly higher level of satisfaction with practical classes without patients (*U* = 7040.5, *p =* 0.003), practical classes with patients (*U* = 7124.5, *p =* 0.002) and student internships (*U* = 6810, *p =* 0.015).

It was also found that 4th -year students were significantly more content with their practical skills than the 6th -year group, *U* = 6832, *p =* 0.013. Moreover, they presented a higher level of satisfaction with the amount of learning material than both 1st -year students, *U* = 5687.5, *p =* 0.031, and the last-year students, *U* = 6679, *p =* 0.048. Likewise, the middle group was significantly more content with time spent on studies, when compared to the first, *U* = 5687.5, *p =* 0.004, and the last year’s group, *U* = 7048.5, *p =* 0.004.

The results indicated that there were no significant differences between the studied groups in the assessment of satisfaction with the mode of studies (full-time vs. part-time), level of knowledge, extra activity during studies (e.g. scientific, social, artistic, sports), peer relationships (outside the medical studies environment), romantic relationships, relationships with patients and relationships with the medical staff.

### Values

A Fisher’s exact test was performed to evaluate whether the proportions for values chosen by students differed by the year of study (Table 3). Due to multiple comparisons, Benjamini-Hochberg p-value correction was applied.

**Table 3:** Differences in values between 1st, 4th and 6th -year students

Values	*n* (%) of students			*p*
	1st year (*n* = 119)	4th year (*n* = 118)	6th year (*n* = 97)	
Peace and quiet	19 (16%)	45 (38%)	31 (32%)	0.006**
Education	32 (27%)	12 (10%)	18 (19%)	0.021*
Achievement and fame	9 (8%)	2 (2%)	0 (0%)	0.021*

* *p* < 0.05, ** *p* < 0.01, *** *p* ≤ 0.001

The results showed that there was a statistically significant difference between whether students chose peace and quiet as their life value and their year of study; among studied groups, 4th -year students occurred to indicate it most often and 1st -year students– least often (*p* = 0.006). Choice of education as life value differed by the year of study, too; 1st -year students indicated it most often and the 4th -year group– least often (*p* = 0.021). It was also found that there was a significant difference between whether students chose achievement and fame as their life value and their year of study; the 1st -year group indicated it most often, and 6th -year students didn’t indicate it at all (*p* = 0.021).

The results showed that there were no significant differences between the studied groups in indicating a happy family life, good health, integrity, religious faith, prosperity of the nation, being respected, work, freedom to express one’s own opinion, friends, wealth and prosperity, participation in democratic political and social affairs, access to culture (art, music, literature, film) or a life of adventure as their life values.

### Resilience

A Mann-Whitney U test was performed to evaluate whether resilience differed by the year of study. Due to multiple comparisons, Benjamini-Hochberg p-value correction was applied.

The results indicated no significant differences in resilience between 1st, 4th and 6th-year medical students.

### Expected and developed competencies

A Fisher’s exact test was performed to evaluate whether the proportions for competencies chosen by students differed by the year of study (Tables 4 and 5). Due to multiple comparisons, Benjamini-Hochberg p-value correction was applied.

**Table 4:** Differences in expected competencies between 1st, 4th and 6th -year students

Expected competencies	*n* (%) of students			*p*
	1st year (*n* = 119)	4th year (*n* = 118)	6th year (*n* = 97)	
Learning, gaining information and introducing changes	67 (56%)	46 (39%)	58 (60%)	0.021*
Time management and planning	24 (20%)	8 (7%)	13 (13%)	0.038*
Stress management and work-life balance	65 (55%)	65 (55%)	31 (32%)	0.007**

* *p* < 0.05, ** *p* < 0.01, *** *p* ≤ 0.001

**Table 5:** Differences in developed competencies between 1st, 4th and 6th -year students

Developed competencies	*n* (%) of students			*p*
	1st year (*n* = 119)	4th year (*n* = 118)	6th year (*n* = 97)	
Working with others and supporting them	32 (27%)	60 (51%)	27 (28%)	0.004**
Managing conflict and negotiating	6 (5%)	19 (16%)	14 (14%)	0.042*
Analysing and Interpreting	23 (19%)	34 (29%)	37 (38%)	0.038*

* *p* < 0.05, ** *p* < 0.01, *** *p* ≤ 0.001

The results showed that there was a statistically significant difference between whether students chose learning, gaining information and introducing changes as the competency they expected to gain during medical studies and their year of study; among studied groups, 6th -year students occurred to indicate it most often and 4th -year students– least often (*p* = 0.021). It was found that choice of time management and planning as expected competency differed by the year of study, too; the first group chose it most often, and the middle group– least often (*p* = 0.038). What’s more, the results showed that choice of stress management and work-life balance differed by the year of study, as 1st and 4th -year students indicated it more often than 6th -year students (*p* = 0.007).

Significant differences were found regarding already gained competencies as well. The results showed that the choice of working with others and supporting them as competency developed during medical studies differed by the year of study, as 4th -year students indicated it more often than 1st and 6th -year students (*p* = 0.004). There was a significant difference between whether students chose managing conflict and negotiating and their year of study; among studied groups, 4th -year students occurred to indicate it most often and 1st -year students– least often (*p* = 0.042). It was also found that indicating analysing and interpreting as developed competencies differed between the studied groups; 6th -year students chose it most frequently, and 1st -year students– least often (*p* = 0.038).

The results showed that there were no significant differences between the studied groups in indicating decision making and responsibility; working with others and supporting them; managing conflict and negotiating; analysing and interpreting; achieving goals and ambition as competencies they expected to gain during medical studies.

Likewise, there were no significant differences between the studied groups in indicating decision making and responsibility; learning, gaining information and introducing changes; time management and planning; stress management and work-life balance; achieving goals and ambition as competencies they developed during medical studies.

### Health and stress level assessment

A Mann-Whitney U test was performed to evaluate whether assessment of students’ health and current stress level associated with studies differed by the year of study (Table 6). Due to multiple comparisons, Benjamini-Hochberg p-value correction was applied.

**Table 6:** Differences in health and stress level assessment between 1st, 4th and 6th -year students

	Median (IQR)			*U*	*p*
	1st year (*n* = 119)	4th year (*n* = 118)	6th year (*n* = 97)		
Health assessment	5 (5–6)		6 (5–6)	4691	0.039*
Stress level assessment	3 (2–5)	5 (3–6)		4613	0.000***
		5 (3–6)	3 (2–5)	7962.5	0.000***

* *p* < 0.05, ** *p* < 0.01, *** *p* ≤ 0.001, IQR - interquartile range

The results indicated that 1st -year students assessed their health significantly lower than 6th -year students, *U* = 4691, *p* = 0.039. It was also found that 4th -year students assessed their stress levels significantly higher than both 1st -year students, *U* = 4613, *p* < 0.001, and 6th -year students, *U* = 7962.5, *p* < 0.001.

Moreover, the results indicated significant differences between the assessment of current stress level and the stress level in previous years for both 4th (*U* = 10990, *p* < 0.001) and 6th -year students (*U* = 5469, *p =* 0.046) (Table 7).

**Table 7:** Differences between current stress level and stress level in previous years for 4th and 6th -year students

	Median (IQR)		*U*	*p*
	Current stress level	Stress level in previous years		
4th year (*n* = 118)	5 (3–6)	3 (1–3)	10,990	0.000***
6th year (*n* = 97)	3 (2–5)	3 (1–5)	5469	0.046*

* *p* < 0.05, ** *p* < 0.01, *** *p* ≤ 0.001, IQR - interquartile range

The results indicated that there were no significant differences between the studied groups in the assessment of financial situation and assessment of general life satisfaction.

### Preferred medical career paths

**Fig. 2:**
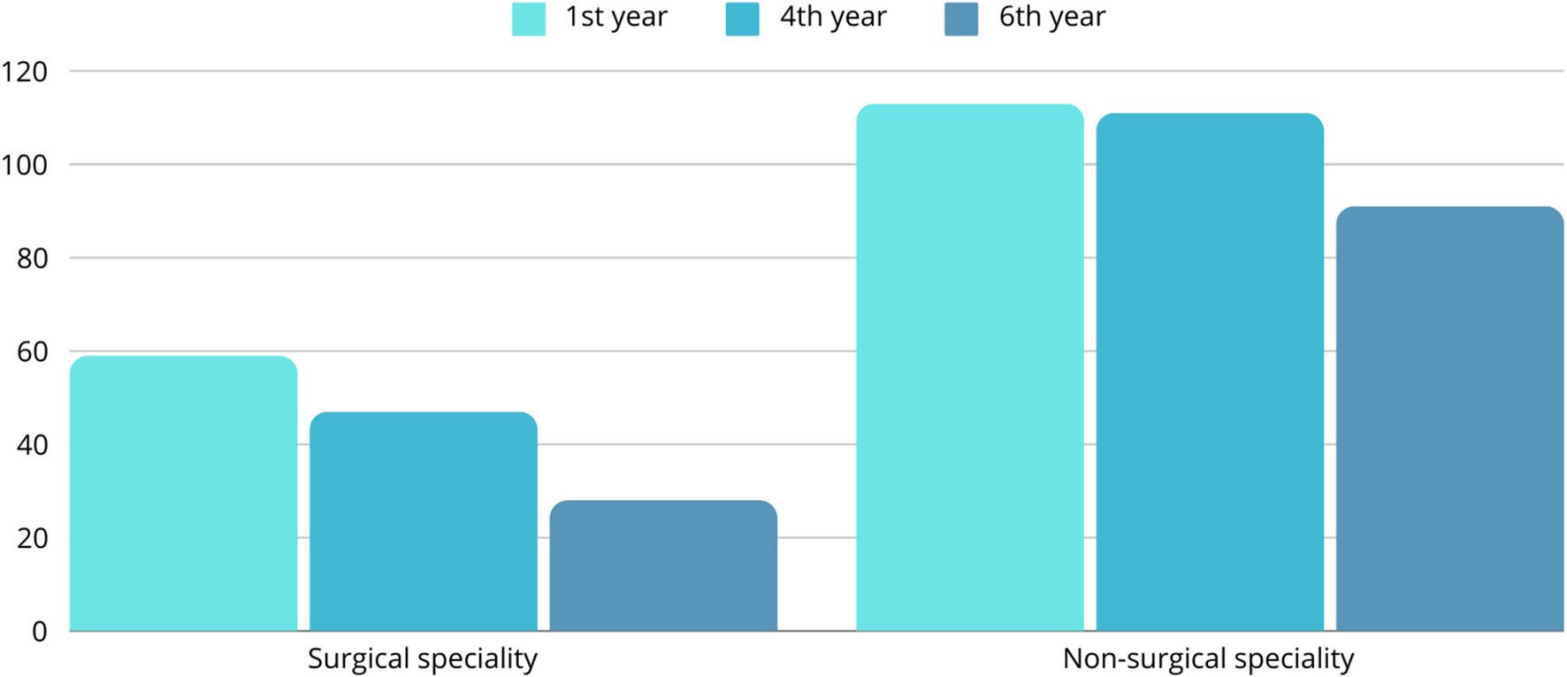
Preferred medical specialties

Participants were asked about their preferred medical career paths - they could choose up to three medical specialties from the list of specialties recognized by the Polish Ministry of Health. All the answers were assigned to the surgical specialty category or the non-surgical specialty category [see Supplementary Material 5]. Figure 2 shows the distribution of participants’ responses.

A Fisher’s exact test was performed to evaluate whether the proportions of the number of types of medical specialties chosen by students differed by the year of study (Table 8). Due to multiple comparisons, Benjamini-Hochberg p-value correction was applied.

**Table 8:** Differences in the number of chosen types of medical specialties between 1st, 4th and 6th -year students

	*n* (%) of students			*p*
	1st year (*n* = 119)	4th year (*n* = 118)	6th year (*n* = 97)	
Both surgical and non-surgical specialty	53 (45%)	40 (34%)	22 (23%)	0.003**

* *p* < 0.05, ** *p* < 0.01, ***p ≤ 0.001

The results showed that there was a statistically significant difference between whether students chose both a surgical and a non-surgical specialty and their year of study– 6th -year students declared such a combination less often than students from two other studied groups (*p* = 0.003).

What is more, 6th-year students were asked about their preferred medical specialties at the beginning of their medical education. These answers were compared to their current choices.

A Fisher’s exact test was performed to evaluate whether the proportions of the number of students who resigned from their original specialty choice differed by the type of medical specialty (surgical or non-surgical) (Table 9). The results showed that there was a statistically significant difference between whether students resigned and the type of specialty– 6th-year students changed their initial choice more often if it was a surgical specialty (*p* = 0.008).

**Table 9:** Differences in the number of resignations between surgical and non-surgical specialties

	*n* (%) of 6th year students who chose		*p*
	surgical specialty (*n* = 28)	non-surgical specialty (*n* = 91)	
Resignation	22 (79%)	45 (49%)	0.008**

* *p* < 0.05, ** *p* < 0.01, *** *p* ≤ 0.001

### Alternatives to studying medicine

**Fig. 3:**
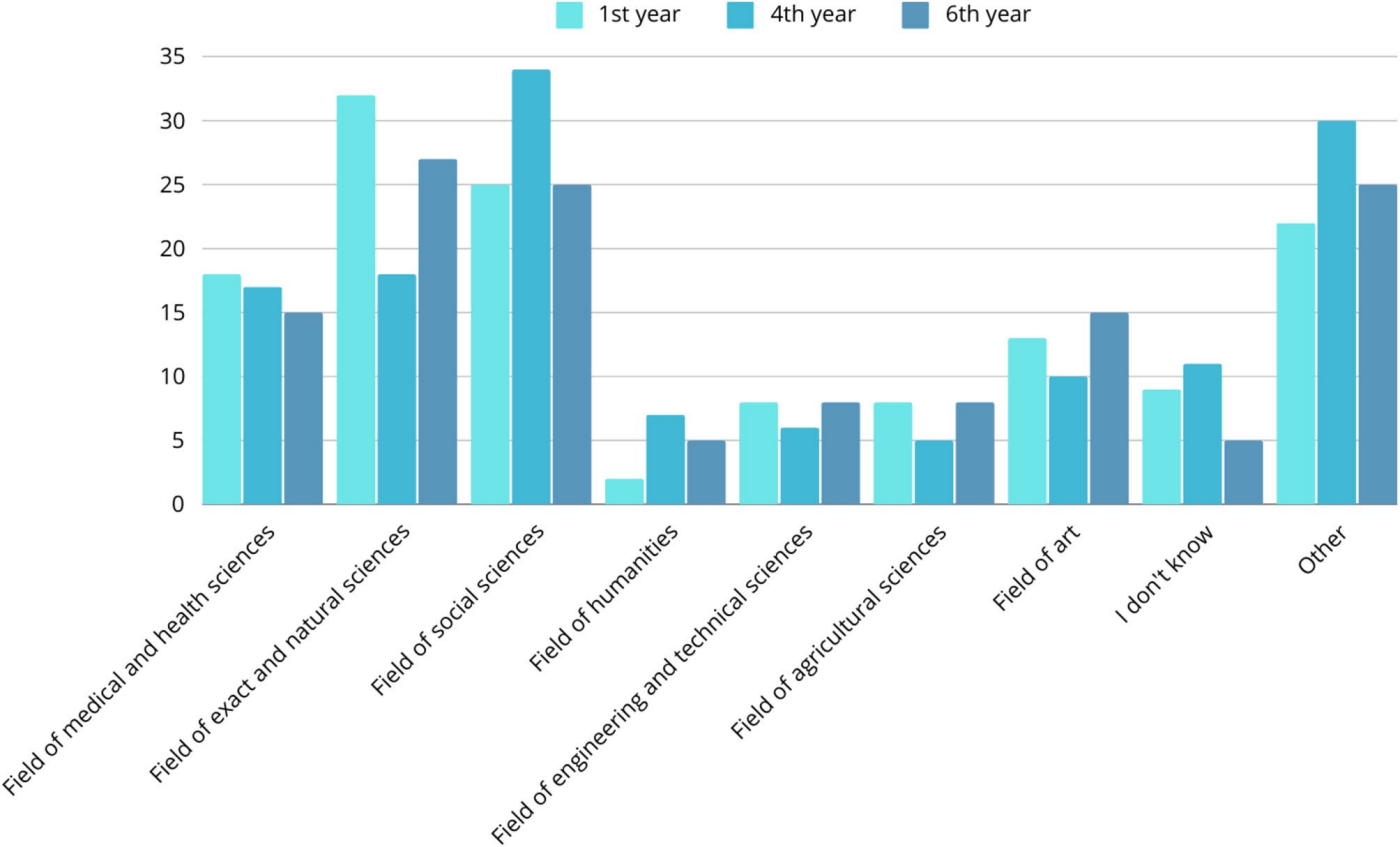
Alternatives to studying medicine

At the end of the survey, students were asked about alternative paths to studying medicine that they might have chosen. The answers were analysed based on the classification of scientific disciplines proposed by the Ministry of National Education in Poland [see Supplementary Material 6]. Figure 3 shows the distribution of participants’ responses.

A Fisher’s exact test was performed to evaluate whether the proportions of alternatives to studying medicine chosen by students differed by the year of study (Table 10). Due to multiple comparisons, Benjamini-Hochberg p-value correction was applied.

**Table 10:** Differences in alternatives to studying medicine between 1st, 4th and 6th -year students

Alternative to studying medicine	*n* (%) of students			*p*
	1st year (*n* = 119)	4th year (*n* = 118)	6th year (*n* = 97)	
Field of exact and natural sciences	32 (27%)	17 (14%)	27 (28%)	0.036*

* *p* < 0.05, ** *p* < 0.01, ***p ≤ 0.001

The results showed that there was a statistically significant difference between whether students chose the field of exact and natural sciences as their alternative to studying medicine and their year of study– 4th -year students occurred to indicate it least often (*p* = 0.036).

A Fisher’s exact test was performed to evaluate whether the variety of chosen alternatives to studying medicine differed by the year of study (Table 11). Due to multiple comparisons, Benjamini-Hochberg p-value correction was applied. As there was no possibility to match all answers to the distinguished fields of science, some answers were excluded from the analysis.

**Table 11:** Differences in number of chosen alternatives to studying medicine between 1st, 4th and 6th -year students

	*n* (%) of students			*p*
	1st year (*n* = 91)	4th year (*n* = 85)	6th year (*n* = 76)	
More than one field of sciences	16 (18%)	18 (21%)	28 (37%)	0.023*

* *p* < 0.05, ** *p* < 0.01, *** *p* ≤ 0.001

The results showed that there was a statistically significant difference between whether students chose more than one field of sciences as their alternative to studying medicine and their year of study– 6th -year students declared more varied alternatives than students from two other studied groups (*p* = 0.023).

## Discussion

The study managed to identify statistically significant differences between the groups of medical students in their first, fourth and sixth year of study. These differences were visible in the fields of: overall satisfaction with the course and its specific components, motivations to study medicine, health and stress assessment, life values, and skills both expected to develop and already gained in the course of medical studies. Based on these differences, a characterisation of the studied groups can be constructed.

First-year medical students have a significantly higher level of overall satisfaction with their studies than their 4th and 6th-year colleagues. This may also be due to merely being freshly accepted into the university, especially that 1st-year students also are the most satisfied with their particular university of choice. High satisfaction may also reflect initial enthusiasm and unverified expectations toward the university and the course - research suggests that 1st-year medical students have specific expectations towards their studies that aren’t later confirmed by reality [31]. Despite the fact that the above-mentioned factors can be a source of stress as well [32], there is evidence that 1st-year students tend to improve their coping effectiveness with time, which in turn translates to improved health and mood [17].

Taking a closer look at the specifics of the high satisfaction level, 1st-year students are more content with their relationships with other medical students, which is perhaps a counterintuitive finding since one could assume that people grow closer to each other in the course of their studies - possibly, it is the growing competition and rivalry that turn this around, or - the decrease in benevolence and conformity as the studies progress [2]. On the other hand, it is possible that 1st-year students see their relationships with peers as more of a priority than older students in order to adapt to their new circumstances, as high stress levels have been found to be less prevalent among students who engage in social activities, such as the student activities club [32]. Additionally, 1st-year students express great satisfaction with their relationships with teachers and are more content with both theoretical and practical classes without contact with a patient. This may be due to the preclinical years’ emphasis on coursework over patient contact or differing values and expectations among 1st-year students. They were the most frequent group of students to choose education as one of their most important life values and the least frequent to choose peace and quiet, which may indicate that they are, in fact, content with the significant amount of material to learn provided by a number of theoretical classes and they like keeping themselves busy - especially that among the groups of students they also more frequently claimed to expect to work on their time management and planning skills, stress management skills and work-life balance during their studies. After all, the fulfilment of aspirations remains one of the most frequently reported motives for pursuing a medical degree among Polish students [5] and students who participate in extracurricular or research activities have been found to be less emotionally exhausted than those who do not [32], perhaps indicating that having more responsibilities does not have to equal greater distress. The first years of medical studies require learning lots of information in a short period of time and 1st-year medical students list ‘enjoying the personal challenge and variety of medicine’ as one of their hopes for the course [33]. In the current study, they were at the same time least likely to choose conflict management, negotiating and analysis and interpretation skills as the ones they had gained during their 1st-year of studying - skills that are in fact mostly developed when cooperating with other doctors or patients and using one’s knowledge in the clinical field [34]. This corresponds to being focused on theoretical and practical classes without patients at the beginning of medical studies. 1st-year students were also most likely to choose achievement and fame as their values, as well as potential high income and social prestige as their motivators, which perhaps also explains their satisfaction levels - associating starting their journey through the medical course with getting closer to fulfilling these values and keeping them in mind as a motivator when being met with high learning requirements, while at the same time still missing the perspective given in the course of the studies.

On top of these findings, significant differences in motivation were found between first-year students and those in later years. First-year students more frequently indicated interest as a key motivator, which may relate to their expectations towards medical studies and their recent final exams. However, a study by Del-Ben et al. shows a significant decline in intrinsic motivation during the first year [7], possibly leading to reduced interest-driven motivation in later years. Interest, passion and ambition have been found to be among the least frequently reported motives for pursuing a medical career among Polish students in their final year [6].

One finding inconsistent with high overall satisfaction among 1st-year medical students is their own health assessment, which is lower than that of 6th-year students, whether it be due to a considerably lower level of knowledge of the human body in the first year or confronting one’s own definition of being healthy with the problems that patients face in the later years of medical education. Interestingly enough, Srivastava et al. find a significant rise in the number of physiological complaints among medical students with time [18]. However, Wolf and Kissling argue that during the 1st year of medical education students’ scores tend to decrease in the areas of physical activity, sleep, a balanced diet and general health [17], which is perhaps more consistent with the findings of this study.

Medical students in their 4th year tend to have lower levels of overall course satisfaction when compared to those in the 1st year. They are less content with their university of choice, theoretical classes and practical classes without patients. Most importantly, they report the highest overall stress levels among the 3 studied groups of students - a finding refuted by Park et al. stating that stressed students are more likely to be younger, in their 1st year of medical education, whose initial years prove the most stressful [15]. The authors, however, agree that these are slightly controversial results. 4th-year students have previously been found to value support more than 1st-year students [2]. It is perhaps 4th-year students’ first encounter with working clinically with patients that is the reason for the stress levels, as well as the decreasing satisfaction with certain components of the medical course. 4th-year students may struggle the most with adapting to the clinical setting of their studies, which was confirmed by Compton et al. in finding that students transitioning to clinical care experience more stress and depressive symptoms than 1st-year students [16]. Some stressors listed by medical students and professionals, such as contact with suffering patients, fear of doing harm or health hazard exposure [12, 13] first become relevant when transitioning to bedside teaching. In the current study this is further reflected by 4th-year students judging their present stress levels as significantly higher than those in their previous years of education.

There are, however, certain aspects, with which 4th-year students are more content than any other group, that being the amount of learning material and the time spent on studying, even though they have been found to report time limitation for training as one of their top stressors [13]. At the same time, they report peace and quiet most often and education least often as their values, a situation quite reversed when juxtaposed with the results for 1st-year students. This leads one to believe that 4th-year students, having gained the experience of the first 3 non-clinical years of medical education, see their priorities not in learning per se, but rather in maintaining a healthy work-life balance and fostering their relationships with other people - they tend to choose stress management and work-life balance as skills they expect to develop. This is reflected in further findings − 4th-year students are least likely to choose learning, gaining information and introducing changes, and time management and planning as competencies they would like to obtain. Perhaps they don’t expect to gain these skills anymore, since they assume they have already acquired them in the pre-clinical years of study. At the same time, they are most likely to indicate working with others and supporting them, as well as managing conflict and negotiating as skills they have already developed, perhaps through their first clinical classes - according to Wright et al. they are significantly more confident about communicating with patients than 1st-year students despite a lack of difference in knowledge thereof [35]. What is more, 4th-year students are, interestingly enough, least likely to claim that they would be pursuing education in exact and natural sciences, were they not involved in studying medicine, which seems consistent with their previously mentioned priorities.

It is also important and perhaps surprising to note that 4th-year students are more content with their practical skills, and thus also with practical classes both with and without patients and internships than 6th-year students, which might be due to a sudden gain in practical abilities in their first clinical year, as well as a temporary lack of an approaching deadline of putting these skills to use as a profession. This is confirmed by findings that defective skills or fear of doing harm to patients are much more of a stressor for final year students than for those in their 4th year of medical education [13], but stands in contrast with studies reporting that 4th-year students experience an increase in imposter feelings [21].

In their final year, medical students report the lowest overall satisfaction, particularly with their university and peer relationships. Surprisingly, despite shared experiences, 6th-year students are less content than 1st-years, possibly due to the desire for change or increasing competition. Not only that, they also have less satisfying relationships with their tutors than students of the 4th year, having perhaps to do with their lessened satisfaction with theoretical classes, as well as practical’s with and without patients, and internships. These findings are consistent with existing research - students in their final clinical year have been found to report relationship, hospital and professional problems, such as worry about the future or high parental expectations, more frequently than students in previous clinical years [13]. They are also prone to higher cynicism [32]. 6th-year students are not as content with their practical skills as 4th-year students, which might be caused by a certain insecurity that stems from being soon confronted with becoming a doctor and not feeling fully prepared to be one. In fact, many medical students experience imposter feelings, which include persistent self-doubt and may impact their confidence negatively [19] - even though studies have shown that these imposter feelings seem to peak around the 3rd year of medical school [20]. 6th-year students do, however, list ‘defective clinical practice skills’ as one of their top stressors [13]. Additionally, 6th-year students report lower stress levels in their previous years of studies as compared to the currentones, which is consistent with findings showing that high stress and emotional exhaustion are more prevalent in this group than in previous clinical years [13].

In addition to that, 6th-year students do not report their interests as their motivation to study as frequently as younger students. This poses a question of whether they confronted their expectations for the scope of the course with reality or maybe lost part of their interest in medicine - interestingly enough, they tend to report alternatives to studying medicine that belong to various fields of science. Based on our results students seems to have relatively consistent ideas about them, being least likely to choose their preferred specialty from both surgical and non-surgical ones - which they probably tend to, as they seem to give up on a surgical specialty more often than on a non-surgical specialty. What is interesting, is that 6th-year students do not indicate achievement and fame as their life values at all, which is not reflected in previous research - according to Kusurkar et al. extrinsic motivations like finances or societal status may increase over time, as opposed to intrinsic motivation that tends to decline [8]. However, at the end of their studies 6th-year students may have reconnected with their intrinsic goals in medicine through the experience with patients or may be too burnt-out to consider these things as important anymore - as their tendency to burn out increases with the progression of the course [11]. An interesting comparison to the results of this research is also provided by a study by Waszkiewicz et al., where final-year medical students most frequently report the desire to help others as their motive to study medicine, followed by employment security and prestige, with passion and interest among the least frequently mentioned motivators [6]. They are most likely to indicate learning, gaining information and introducing changes as skills expected to be gained, perhaps feeling like they lack the necessary competencies [13] and now is their last opportunity to develop these. On the other hand, they are least likely to expect a gain in stress management and work-life balance skills, having supposedly had enough time to already acquire these throughout the years. Similarly, they most frequently report analysis and interpretation as skills they have already gained, which may indicate that it is, indeed, the clinical years that have contributed to this the most.

### Limitations of the study and future research directions

This study’s cross-sectional design limits the ability to observe how individual students’ motivation, values, satisfaction, competencies, and stress levels change over time; future research should therefore adopt a longitudinal design to track these factors across different stages of medical education, allowing for the differentiation between developmental and external influences. The low internal consistency found in the post-hoc validation of the motivation questionnaire suggests that it does not sufficiently capture the complexity of students’ motives for studying medicine; subsequent studies should prioritize the improvement and revalidation of this instrument to ensure more reliable and meaningful assessments. The exclusive focus on Polish medical students restricts the generalizability of the findings to other cultural and educational contexts; expanding future studies to include international cohorts would enhance the applicability of results and enable cross-cultural comparisons. Additionally, recruitment through social media and university emails may have led to selection bias, potentially skewing the sample toward more engaged or digitally active students; future research should consider employing broader and more randomized recruitment strategies. The reliance on self-reported data introduces the risk of social desirability bias, whereby students may provide responses that reflect perceived social norms rather than their true experiences; incorporating qualitative methods or third-party evaluations could help mitigate this issue. Two promising areas for future investigation include also exploring the underlying causes of the observed decline in interest-driven learning and the valuing of achievement and fame across medical training, as well as identifying the factors that shape the quality of relationships between students and tutors—an element likely to play a significant role in shaping both academic outcomes and professional identity formation.

## Conclusions

Motivations of students to learn progressively assume a less extrinsic direction related to income and prestige, but interest-driven learning seems to decrease as well. Overall, student satisfaction with medical education seems to decline as the course progresses, in terms of areas such as university, relationships, classes or skills. The highest satisfaction with the amount of work concerns the 4th year. Values such as achievement and fame wear off with time, but so does valuing education, making space for peace and quiet. There are no significant differences in resilience between the students of the 1st, 4th and 6th year. Interpersonal skills seem to develop in the later stages of education, same as analytical skills. Expectations regarding obtaining specific skills range from planning- and time-management-oriented at the beginning to work-life balance in the middle, and learning-related at the end of the studies. Stress levels tend to increase in the course of the studies, though their reasons vary. The transition into clinical practice, however, seems to be crucial when explaining the highest stress levels reported by 4th-year students. Few results have been obtained regarding the changes in specialty or alternative educational path preference. The least likely group of students to pursue an alternative path in exact and natural sciences are 4th-year students, while 6th-year students report the greatest variety of ideas for alternative pathways. Among 6th-year students, ideas to pursue a surgical specialty are more frequently abandoned than a non-surgical specialty and the two types of specialties are rarely reported together as potential future career paths.

## Electronic supplementary material

Below is the link to the electronic supplementary material.

Supplementary Material 1

## Data Availability

Due to the specific nature of the psychological variables studied, the data collected in this research are not publicly available. However, interested parties can request access to the data by contacting the authors directly.

## Abbreviations

BRS: Brief Resilience Scale
CBOS: Public Opinion Research Centre (Centrum Badania Opinii Społecznej)
IQR: interquartile range
