# Supplementary material for: Assessing the impact of medical studies on students’ motivation, satisfaction, stress and values in Poland: a cross-sectional study
Source: BMC Med Educ. 2025 May 16;25:714. doi: 10.1186/s12909-025-07287-4 (PMC12085074; doi:10.1186/s12909-025-07287-4)
Supplement: Supplementary file 1 — Supplementary Material 1 [file 12909_2025_7287_MOESM1_ESM.docx]

**SUPPLEMENTARY MATERIAL 1**

**Evaluation of personal circumstances questionnaire**

Rate the following aspects of your personal situation:

|  | Very bad | Bad | Rather bad | I have no opinion | Rather good | Good | Very good |
| --- | --- | --- | --- | --- | --- | --- | --- |
| Your financial situation |  |  |  |  |  |  |  |
| Your health |  |  |  |  |  |  |  |
| Life satisfaction in general |  |  |  |  |  |  |  |
| Current levels of stress related to your studies |  |  |  |  |  |  |  |
| Stress related to your studies in the previous years (years 4 and 6) |  |  |  |  |  |  |  |

**SUPPLEMENTARY MATERIAL 2**

**Motivation to study medicine questionnaire**

Rate the extent, to which you agree with the following statements:

Choosing to study medicine was:

|  | I definitely disagree | I disagree | I rather disagree | I have no opinion | I rather agree | I agree | I definitely agree |
| --- | --- | --- | --- | --- | --- | --- | --- |
| My conscious decision |  |  |  |  |  |  |  |
| A decision made based on others’ expectations towards me |  |  |  |  |  |  |  |

I chose to study medicine primarily because of:

|  | I definitely disagree | I disagree | I rather disagree | I have no opinion | I rather agree | I agree | I definitely agree |
| --- | --- | --- | --- | --- | --- | --- | --- |
| A clear and secure future |  |  |  |  |  |  |  |
| My interests |  |  |  |  |  |  |  |
| High income in the future |  |  |  |  |  |  |  |
| Social prestige |  |  |  |  |  |  |  |
| Knowledge useful in private life |  |  |  |  |  |  |  |
| My family members work in healthcare |  |  |  |  |  |  |  |
| I want to feel needed by others |  |  |  |  |  |  |  |

Are you studying medicine for any other reason not listed above?

…

**Validation of motivation to study medicine questionnaire**

As motivation to study medicine questionnaire was created by the research team, its partial validation was carried out. Due to the fact that this was a post-factum validation, certain stages of typical analysis couldn’t be performed.

To evaluate the internal consistency of the questionnaire, the Cronbach-alpha coefficient and the McDonald’s-omega reliability coefficient were used. The exploratory factor analysis was performed in order to confirm its unidimensionality. The results of the motivation questionnaire in studied groups are presented in Table 1.

**Table 1** The results of motivation to study medicine questionnaire in studied groups

| **Items** | **Median (IQR)** | | |
| --- | --- | --- | --- |
|  | **1st year students** | **4th year students** | **6th year students** |
| My conscious decision | 7 (6-7) | 7 (6-7) | 7 (5-7) |
| A decision made based on others’ expectations towards me | 2 (1-3) | 2 (1-3) | 2 (1-5) |
| A clear and secure future | 6 (5-7) | 6 (5-7) | 6 (5-7) |
| My interests | 7 (6-7) | 6 (5.25-7) | 6 (5-7) |
| High income in the future | 6 (5-7) | 5 (5-6) | 6 (5-6) |
| Social prestige | 5 (3-6) | 5 (3-6) | 5 (3-6) |
| Knowledge useful in private life | 6 (4.5-7) | 5 (4-6) | 6 (5-7) |
| My family members work in healthcare | 1 (1-3) | 1 (1-3) | 1 (1-5) |
| I want to feel needed by others | 6 (4-7) | 6 (5-7) | 6 (4-6) |

IQR - interquartile range

The Cronbach-alpha coefficient and the McDonald’s-omega reliability coefficient for motivation questionnaire are displayed in Table 2. The values obtained with both methods suggest low internal consistency of the questionnaire in its original version.

**Table 2** Reliability analysis results – original version of questionnaire

|  | **Cronbach-alpha** | **McDonald’s-omega** |
| --- | --- | --- |
| Motivation to study medicine questionnaire | 0.54 | 0.13 |

Table 3 shows item-total correlation and how Cronbach-alpha coefficient changes after excluding each item. Presented results suggest that particular items should be excluded in order to improve reliability of the motivation questionnaire.

**Table 3** Item-total correlation and Cronbach-alpha coefficient changes – original version of questionnaire

| **Items** | **Item-total correlation** | **Cronbach-alpha after excluding item** |
| --- | --- | --- |
| My conscious decision | 0.21 | 0.54 |
| A decision made based on others’ expectations towards me | - 0.15 | 0.59 |
| A clear and secure future | 0.6 | 0.52 |
| My interests | 0.38 | 0.53 |
| High income in the future | 0.54 | 0.56 |
| Social prestige | 0.6 | 0.52 |
| Knowledge useful in private life | 0.52 | 0.52 |
| My family members work in healthcare | 0.16 | 0.62 |
| I want to feel needed by others | 0.27 | 0.56 |

For further analysis, two items were removed. The Cronbach-alpha coefficient and the McDonald’s-omega reliability coefficient for improved version of motivation questionnaire are displayed in Table 4. The values obtained with both methods suggest moderate internal consistency of the questionnaire.

**Table 4** Reliability analysis results – improved version of questionnaire

|  | **Cronbach-alpha** | **McDonald’s-omega** |
| --- | --- | --- |
| Motivation to study medicine questionnaire | 0.62 | 0.6 |

Table 5 shows item-total correlation and how Cronbach-alpha coefficient changes after excluding each item in the improved version of questionnaire. All correlations are >0,3, which suggests leaving all questions within the questionnaire is justified.

**Table 5** Item-total correlation and Cronbach-alpha coefficient changes – improved version of questionnaire

| **Items** | **Item-total correlation** | **Cronbach-alpha after excluding item** |
| --- | --- | --- |
| My conscious decision | 0.34 | 0.62 |
| A clear and secure future | 0.55 | 0.56 |
| My interests | 0.49 | 0.59 |
| High income in the future | 0.48 | 0.59 |
| Social prestige | 0.55 | 0.55 |
| Knowledge useful in private life | 0.48 | 0.57 |
| I want to feel needed by others | 0.31 | 0.63 |

Assessment of unidimensionality of the improved version of motivation questionnaire is displayed in Table 6. As presented results indicated the possibility of improving model fit, further factor analysis was performed. In order to confirm whether any particular factors could be detected within the questionnaire, two additional models were created. The Cronbach-alpha coefficients and the McDonald’s-omega reliability coefficients for each model and its factors are displayed in Table 7.

**Table 6** Assessment of unidimensionality of motivation to study medicine questionnaire

| **Unidimen-sionality index** | **tau** | **Cronbach-alpha** | **Correlation mean*** | **Correlation median**** | **CFI** | **ECV** |
| --- | --- | --- | --- | --- | --- | --- |
| 0.36 | 0.57 | 0.62 | 0.2 | 0.17 | 0.55 | 0.45 |

*mean of correlations between items

**median of correlations between items

CFI - comparative fit index

ECV - explained common variance

**Table 7** Reliability analysis for tested models

| **Model** | **Factor** | **Cronbach-alpha** | **McDonald’s-omega** |
| --- | --- | --- | --- |
| 1 | 1 | 0.69 | 0.71 |
|  | 2 | 0.54 | 0.55 |
| 2 | 1 | 0.67 | - |
|  | 2 | 0.67 | - |
|  | 3 | 0.45 | 0.46 |

The model with two factors proved to have the highest reliability coefficients when compared to one factor- and three factors models. Table 8 displays its further analysis.

**Table 8** Assessment of unidimensionality of each factor in two factors model of motivation questionnaire

| **Factor** | **Unidimen-sionality index** | **tau** | **Cronbach-alpha** | **Correlation mean*** | **Correlation median**** | **CFI** | **ECV** |
| --- | --- | --- | --- | --- | --- | --- | --- |
| 1 | 0.96 | 0.96 | 0.69 | 0.48 | 0.52 | 1 | 0.96 |
| 2 | 0.78 | 0.8 | 0.54 | 0.26 | 0.2 | 0.98 | 0.82 |

*mean of correlations between items

**median of correlations between items

CFI - comparative fit index

ECV - explained common variance

Analysis performed on the improved version of motivation to study medicine questionnaire suggests it contains hidden constructs. The exploratory factor analysis showed a possibility to extract two factors which may refer to external and intrinsic motivation (Table 9).

**Table 9** Model with two factors – item details

| **Factor** | **Item** |
| --- | --- |
| 1 – external motivation | A clear and secure future |
|  | High income in the future |
|  | Social prestige |
| 2 – intrinsic motivation | My conscious decision |
|  | My interests |
|  | Knowledge useful in private life |
|  | I want to feel needed by others |

**SUPPLEMENTARY MATERIAL 3**

**Satisfaction with studying medicine questionnaire**

Rate your satisfaction with the following areas of studying. If you have not had experience in some of them yet, select the "not applicable" option.

|  | very dissatisfied | dissatisfied | partly dissatisfied | I have no opinion | partly satisfied | satisfied | very satisfied | not applicable |
| --- | --- | --- | --- | --- | --- | --- | --- | --- |
| Overall satisfaction with medical studies |  |  |  |  |  |  |  |  |
| The mere fact of being admitted to medical studies |  |  |  |  |  |  |  |  |
| Studying at your current university |  |  |  |  |  |  |  |  |
| Mode of studies - full-time/part-time |  |  |  |  |  |  |  |  |
| My knowledge level |  |  |  |  |  |  |  |  |
| My practical skills |  |  |  |  |  |  |  |  |
| Extra activity during studies (e.g. scientific, social, artistic, sports) |  |  |  |  |  |  |  |  |
| The amount of learning material |  |  |  |  |  |  |  |  |
| Time spent on studies |  |  |  |  |  |  |  |  |
| Relationships with other medical students |  |  |  |  |  |  |  |  |
| Peer relationships (outside the medical studies environment) |  |  |  |  |  |  |  |  |
| Romantic relationships |  |  |  |  |  |  |  |  |
| Relationships with patients |  |  |  |  |  |  |  |  |
| Relationships with lecturers |  |  |  |  |  |  |  |  |
| Relationships with the medical staff (apart from those who hold classes) |  |  |  |  |  |  |  |  |
| Theoretical classes - lectures, seminars |  |  |  |  |  |  |  |  |
| Practical classes without patients - practicals, laboratories |  |  |  |  |  |  |  |  |
| Practical classes with patients |  |  |  |  |  |  |  |  |
| Student internships |  |  |  |  |  |  |  |  |

**Validation of satisfaction with studying medicine questionnaire**

As satisfaction with studying medicine questionnaire was created by the authors, its partial validation was carried out. Due to the fact that this was a post-factum validation, certain stages of typical analysis couldn’t be performed.

To evaluate the internal consistency of the questionnaire, the Cronbach-alpha coefficient and the McDonald’s-omega reliability coefficient were used. The exploratory factor analysis was performed in order to confirm its unidimensionality. The results of the satisfaction questionnaire in studied groups are presented in Table 1.

**Table 1** The results of satisfaction with studying medicine questionnaire in studied groups

| **Items** | **Median (IQR)** | | |
| --- | --- | --- | --- |
|  | **1st year students** | **4th year students** | **6th year students** |
| Overall satisfaction with medical studies | 6 (5-6.5) | 5 (3-6) | 5 (2-6) |
| The mere fact of being admitted to medical studies | 7 (6-7) | 6.5 (6-7) | 7 (6-7) |
| Studying at current university | 6 (6-7) | 6 (5-7) | 5 (3-6) |
| Mode of studies (full-time vs part-time) | 6 (6-7) | 7 (5.25-7) | 6 (5-7) |
| Level of knowledge | 5 (4-6) | 5 (3-6) | 5 (3-5) |
| Practical skills | - | 3 (2-5) | 3 (1-5) |
| Extra activity during studies (e.g. scientific, social, artistic, sports) | 4 (3-6) | 5 (3-6) | 5 (3-6) |
| Amount of learning material | 3 (2-5) | 4 (3-5) | 3 (2-5) |
| Time spent on studies | 3 (2-5) | 5 (3-5) | 3 (2-5) |
| Relationships with other medical students | 6 (4-6) | 5 (3-6) | 5 (3-6) |
| Peer relationships (outside the medical studies environment) | 6 (5-7) | 6 (5-6) | 5 (4-6) |
| Romantic relationships | 5 (3-7) | 5 (3-7) | 6 (3-7) |
| Relationships with patients | - | 6 (5-6) | 6 (5-6) |
| Relationships with lecturers | 5 (4-6) | 5 (5-6) | 5 (4-6) |
| Relationships with the medical staff | - | 5 (4-6) | 5 (3-6) |
| Theoretical classes | 5 (4-6) | 5 (3-5) | 4 (2-5) |
| Practical classes without patients | 6 (5-6) | 5 (3-6) | 4 (2-5) |
| Practical classes with patients | - | 5 (4-6) | 5 (3-6) |
| Student internships | - | 5 (4-6) | 5 (3-6) |

IQR - interquartile range

The Cronbach-alpha coefficient and the McDonald’s-omega reliability coefficient for satisfaction questionnaire are displayed in Table 2. The values obtained with both methods are similar and suggest high internal consistency of the questionnaire.

**Table 2** Reliability analysis results

|  | **Cronbach-alpha** | **McDonald’s-omega** |
| --- | --- | --- |
| Satisfaction with studying medicine questionnaire | 0.90 | 0.92 |

Table 3 shows item-total correlation and how Cronbach-alpha coefficient changes after excluding each item. All correlations are >0,3 and Cronbach-alpha coefficient decreases slightly or remains unchanged after items are removed, which suggests leaving all questions within the questionnaire is justified.

**Table 3** Item-total correlation and Cronbach-alpha coefficient changes

| **Items** | **Item-total correlation** | **Cronbach-alpha after excluding item** |
| --- | --- | --- |
| Overall satisfaction with medical studies | 0.77 | 0.88 |
| The mere fact of being admitted to medical studies | 0.45 | 0.89 |
| Studying at current university | 0.68 | 0.89 |
| Mode of studies (full-time vs part-time) | 0.33 | 0.90 |
| Level of knowledge | 0.55 | 0.89 |
| Practical skills | 0.63 | 0.89 |
| Extra activity during studies (e.g. scientific, social, artistic, sports) | 0.38 | 0.90 |
| Amount of learning material | 0.62 | 0.89 |
| Time spent on studies | 0.62 | 0.89 |
| Relationships with other medical students | 0.54 | 0.89 |
| Peer relationships (outside the medical studies environment) | 0.56 | 0.89 |
| Romantic relationships | 0.33 | 0.90 |
| Relationships with patients | 0.59 | 0.89 |
| Relationships with lecturers | 0.62 | 0.89 |
| Relationships with the medical staff | 0.63 | 0.89 |
| Theoretical classes | 0.64 | 0.89 |
| Practical classes without patients | 0.66 | 0.89 |
| Practical classes with patients | 0.70 | 0.89 |
| Student internships | 0.61 | 0.89 |

Assessment of unidimensionality of satisfaction questionnaire is presented in Table 4. As the CFI value indicated the possibility of improving model fit, further factor analysis was performed. In order to confirm whether any particular factors could be detected within the questionnaire, four additional models were created. The Cronbach-alpha coefficients and the McDonald’s-omega reliability coefficients for each model and its factors are displayed in Table 5.

**Table 4** Assessment of unidimensionality of satisfaction with studying medicine questionnaire

| **Unidimen-sionality index** | **tau** | **Cronbach-alpha** | **Correlation mean*** | **Correlation median**** | **CFI** | **ECV** |
| --- | --- | --- | --- | --- | --- | --- |
| 0.77 | 0.84 | 0.9 | 0.32 | 0.29 | 0.61 | 0.72 |

*mean of correlations between items

**median of correlations between items

CFI - comparative fit index

ECV - explained common variance

**Table 5** Reliability analysis for tested models

| **Model** | **Factor** | **Cronbach-alpha** | **McDonald’s-omega** |
| --- | --- | --- | --- |
| 1 | 1 | 0.87 | 0.88 |
|  | 2 | 0.83 | 0.83 |
|  | 3 | 0.87 | - |
| 2 | 1 | 0.83 | 0.84 |
|  | 2 | 0.9 | - |
|  | 3 | 0.88 | - |
|  | 4 | 0.85 | 0.87 |
| 3 | 1 | 0.8 | 0.82 |
|  | 2 | 0.9 | - |
|  | 3 | 0.88 | - |
|  | 4 | 0.85 | 0.87 |
|  | 5 | 0.66 | 0.68 |
| 4 | 1 | 0.87 | 0.87 |
|  | 2 | 0.9 | - |
|  | 3 | 0.88 | - |
|  | 4 | 0.66 | 0.68 |
|  | 5 | 0.64 | 0.65 |
|  | 6 | 0.79 | 0.8 |

The Cronbach-alpha coefficients and the McDonald’s-omega reliability coefficients for all models are lower when compared to the original version with all items put together, which suggests that the presented satisfaction questionnaire has no hidden constructs.

**SUPPLEMENTARY MATERIAL 4**

**Demographic characteristics of participants**

| **Characteristics** |  | ***n* (%)** |
| --- | --- | --- |
| Gender |  |  |
|  | Male | 71 (21%) |
|  | Female | 263 (79%) |
| Age |  | *M* = 22.26, *SD* = 2.63 |
| Place of origin |  |  |
|  | Countryside | 72 (22%) |
|  | City up to 100 000 inhabitants | 109 (33%) |
|  | City up to 100 000 – 200 000 inhabitants | 38 (11%) |
|  | City up to 200 000 – 500 000 inhabitants | 40 (12%) |
|  | City over 500 000 inhabitants | 75 (22%) |
| Marital status |  |  |
|  | Not in a formal relationship | 147 (44%) |
|  | In an informal monogamous relationship | 171 (51.2%) |
|  | In an informal polyamorous relationship | 5 (1.5%) |
|  | Married | 6 (1.8%) |
|  | Prefer not to say | 5 (1.5%) |
| Number of friends |  |  |
|  | Studying medicine | *M* = 3, *SD* = 3 |
|  | Met outside the medical studies environment | *M* = 4, *SD* = 3 |
| Mode of studies |  |  |
|  | Full-time | 284 (85%) |
|  | Part-time | 50 (15%) |
| Year of studies |  |  |
|  | 1^st^ year | 119 (36%) |
|  | 4^th^ year | 118 (35%) |
|  | 6^th^ year | 97 (29%) |
| University |  |  |
|  | Medical University of Gdansk | 117 (35%) |
|  | Poznan University of Medical Sciences | 87 (26%) |
|  | Medical University of Lodz | 33 (9.9%) |
|  | Medical University of Warsaw | 19 (5.7%) |
|  | Medical University of Silesia | 13 (3.9%) |
|  | Medical University of Lublin | 9 (2.7%) |
|  | Medical University of Wroclaw | 8 (2.4%) |
|  | Ludwik Rydygier Collegium Medicum in Bydgoszcz (Nicolaus Copernicus University in Toruń) | 7 (2.1%) |
|  | Jagiellonian University | 6 (1.8%) |
|  | Casimir Pulaski Radom University | 6 (1.8%) |
|  | Medical University of Bialystok | 5 (1.5%) |
|  | College of Medical Sciences (University of Rzeszow) | 5 (1.5%) |
|  | Pomeranian Medical University | 5 (1.5%) |
|  | University of Opole | 3 (0.9%) |
|  | Andrzej Frycz Modrzewski Krakow University | 2 (0.6%) |
|  | University of Warmia and Mazury | 2 (0.6%) |
|  | Cardinal Stefan Wyszynski University in Warsaw | 2 (0.6%) |
|  | School of Medicine (Jan Kochanowski University) | 1 (0.3%) |
|  | Lazarski University | 1 (0.3%) |
|  | Maria Sklodowska-Curie Medical University in Warsaw | 1 (0.3%) |
|  | University of Zielona Gora | 1 (0.3%) |
|  | Mazovian University in Plock | 1 (0.3%) |

**SUPPLEMENTARY MATERIAL 5**

**The classification of medical specialties**

| **Non-surgical (with a module in Internal Medicine)** | **Surgical (with a module in General Surgery)** |
| --- | --- |
| 1. Allergology 2. Anesthesiology and Intensive Therapy 3. Angiology 4. Audiology and Phoniatrics 5. Respiratory Diseases 6. Internal Medicine 7. Dermatology and Venereology 8. Infectious Diseases 9. Diabetology 10. Endocrinology 11. Paediatric Endocrinology and Diabetology 12. Clinical Pharmacology 13. Gastroenterology 14. Paediatric Gastroenterology 15. Clinical Genetics 16. Geriatrics 17. Haematology 18. Hypertensiology 19. Clinical Immunology 20. Intensive Therapy 21. Cardiology 22. Paediatric Cardiology 23. Aerospace Medicine 24. Maritime and Tropical Medicine 25. Palliative Medicine 26. Occupational Medicine 27. Emergency Medicine 28. Family Medicine 29. Forensic Medicine 30. Sports Medicine 31. Nephrology 32. Neonatology 33. Neurology 34. Paediatric Neurology 35. Clinical Oncology 36. Paediatric Oncology and Haematology 37. Pathomorphology 38. Paediatrics 39. Psychiatry 40. Child and Adolescent Psychiatry 41. Radiology and Diagnostic Imaging 42. Radiation Oncology 43. Rheumatology 44. Rehabilitation Medicine 45. Sexology 46. Clinical Toxicology | 1. Paediatric Surgery 2. Thoracic Surgery 3. Vascular Surgery 4. General Surgery 5. Surgical Oncology 6. Plastic Surgery 7. Maxillofacial Surgery 8. Gynaecologic Oncology 9. Cardiac Surgery 10. Neurological Surgery 11. Ophthalmology 12. Orthopaedics and Traumatology 13. Otorhinolaryngology 14. Paediatric Otorhinolaryngology 15. Obstetrics and Gynaecology 16. Urology |

**SUPPLEMENTARY MATERIAL 6**

**The classification of sciences used for alternatives to medicine analysis**

| **Category** | **Respondents’ answers** | **Number of answers** |
| --- | --- | --- |
| Social sciences | Psychology and Psychotherapy | 30 |
|  | Law | 16 |
|  | Teaching others/education | 14 |
|  | Economics, finance, accounting | 9 |
|  | Management | 4 |
|  | Working in a social organisation (e.g. hospice, foundation) | 3 |
|  | Politics/political sciences | 3 |
|  | Marketing | 3 |
|  | Sociology | 2 |
|  | Human Resources | 1 |
|  | Public Relations | 1 |
|  | International Relations | 1 |
|  | Logistics | 1 |
|  | Journalism | 1 |
|  | International business | 1 |
|  | Relationship management | 1 |

| Exact and natural sciences | Information Technology | 19 |
| --- | --- | --- |
|  | Chemistry and related (including cosmetology) | 17 |
|  | Biotechnology | 15 |
|  | Biology and related | 15 |
|  | Mathematics | 7 |
|  | Physics | 3 |
|  | Oceanography | 2 |
|  | Meteorology | 1 |

| Medical and health sciences | Nursing or midwifery | 11 |
| --- | --- | --- |
|  | Physiotherapy | 8 |
|  | A medical profession in general | 7 |
|  | Nutrition | 7 |
|  | Pharmaceutics | 7 |
|  | Dentistry | 3 |
|  | Paramedics | 2 |
|  | Medical Analytics | 2 |
|  | Genetics | 1 |

| The Arts | Music | 8 |
| --- | --- | --- |
|  | Arts in general | 6 |
|  | Acting | 6 |
|  | Dance | 4 |
|  | Graphic design | 4 |
|  | Floristry | 2 |
|  | Design | 1 |
|  | Fashion design | 1 |
|  | Artistic make-up | 1 |
|  | Scenography | 1 |
|  | Camera operator | 1 |
|  | Painting or art restoration | 1 |
|  | Ceramics, weaving, painting | 1 |
|  | Film school, photography | 1 |

| Technical sciences and engineering | A technical field of study in general | 7 |
| --- | --- | --- |
|  | Architecture | 7 |
|  | Mechanical engineering (machine construction, mechanics) | 5 |
|  | Biomedical engineering | 2 |
|  | Genetic engineering | 1 |
|  | Automatic Control and Robotics | 1 |

| Agricultural sciences | Veterinary medicine | 17 |
| --- | --- | --- |
|  | Horticulture | 2 |
|  | Agriculture | 1 |
|  | Food Technology | 1 |

| Humanities | Language studies | 11 |
| --- | --- | --- |
|  | Writing | 2 |
|  | History | 1 |
|  | Archeology | 1 |
|  | Literature, publishing | 1 |
